# Acute Levetiracetam-Induced Thrombocytopenia in an Anticoagulated Immunocompetent Patient

**DOI:** 10.7759/cureus.13817

**Published:** 2021-03-11

**Authors:** Somto T Nwaedozie, Sheetal Aatrai, Javad Najjar Mojarrab, Abdullah Al Twal

**Affiliations:** 1 Internal Medicine, Marshfield Clinic Health System, Marshfield, USA; 2 Pulmonary and Critical Care Medicine, Marshfield Clinic Health System, Marshfield, USA

**Keywords:** levetiracetam, thrombocytopenia, adverse drug effect, anticoagulation, seizures

## Abstract

Levetiracetam (LEV) is a commonly prescribed anti-seizure medication for the prophylaxis and treatment of focal and generalized seizures. However, a few significant LEV-associated adverse effects have been reported in the literature. Here, we describe a case of significant thrombocytopenia within 24 hours of IV LEV administration for generalized seizures in an anticoagulated immunocompetent patient that completely resolved following discontinuation of the medication. Increased awareness of this uncommon thrombocytopenic side effect of LEV especially in the setting of anticoagulation is important for clinicians providing care to patients with a history of seizures due to the heightened risk of clinically significant bleeding.

## Introduction

Levetiracetam (LEV) is a commonly used antiepileptic medication for the prophylaxis and treatment of focal and generalized seizures due to its efficacy, tolerance, good pharmacokinetic profile, and relatively benign adverse effects [1-3]. In an open-label placebo-controlled study by Abou-Khalil et al., adverse effects observed in 10% or more of users at individualized doses of 1,000-3,000 mg/day were asthenia, nausea, dizziness, somnolence, nervousness, anorexia, psychiatric side effects as well as other tolerable side effects [4].

 Compared to other similarly used anti-seizure medications, severe adverse effects like hepatotoxicity, hemodynamic imbalances, pancytopenia, or significant drug interactions are not usually associated with its use [5-6]. However, thrombocytopenia has been reported with LEV use in several clinical settings and patient populations, oftentimes along with other thrombocytopenia-inducing medications [1, 7-12]. Here, we report a case of acute significant thrombocytopenia that developed following LEV use in the setting of status epilepticus in an anticoagulated immunocompetent patient. Clinicians should consider the potential for this drug-induced change in hematological parameters when selecting anti-seizure medications for patients on anticoagulants.

## Case presentation

A 43-year-old man was admitted to the medical ICU due to severe coronavirus- disease-2019 (COVID-19) pneumonia requiring intubation. The patient had a past medical history of type 2 diabetes, hypertension, and traumatic brain injury following which he was treated several years ago with anti-seizure medications. He was cognitively functional at baseline.

**Figure 1:**
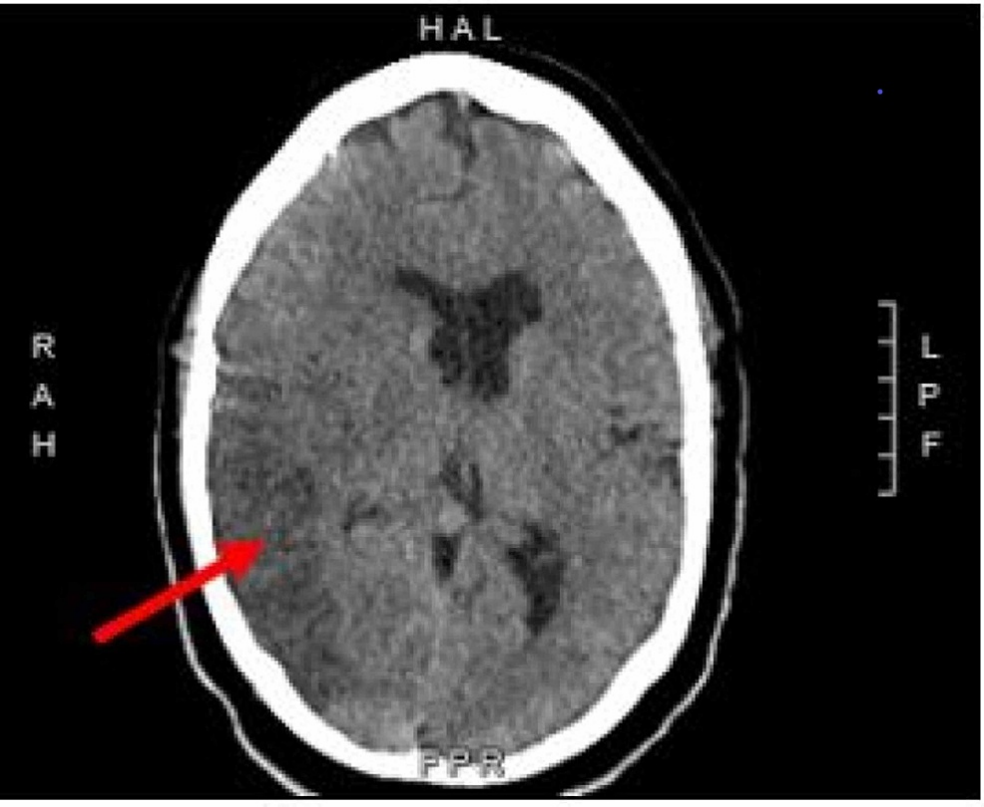
Noncontrast head CT demonstrating a large infarct involving the right MCA vascular distribution with mass effect. MCA, middle cerebral artery

He required endotracheal intubation and mechanical ventilation due to hypoxic respiratory failure and received medical management for SARS-CoV-2 viral pneumonia with convalescent plasma, a 10-day course of remdesivir and dexamethasone, a one-week course of IV piperacillin/tazobactam, and heparin for deep vein thrombosis (DVT) prophylaxis. On the 10th day of hospitalization, the patient was weaned off sedation in anticipation of extubation but was found to be poorly responsive which prompted further diagnostic work-up for cerebral infarction. CT of the head revealed an acute right middle cerebral artery (MCA) infarct with mass-effect within the right cerebral hemisphere (Figure 1). Neurology service was consulted, and the patient started on medical management for right MCA stroke with low dose aspirin, atorvastatin, and 3% normal saline to prevent cerebral edema.

**Figure 2:**
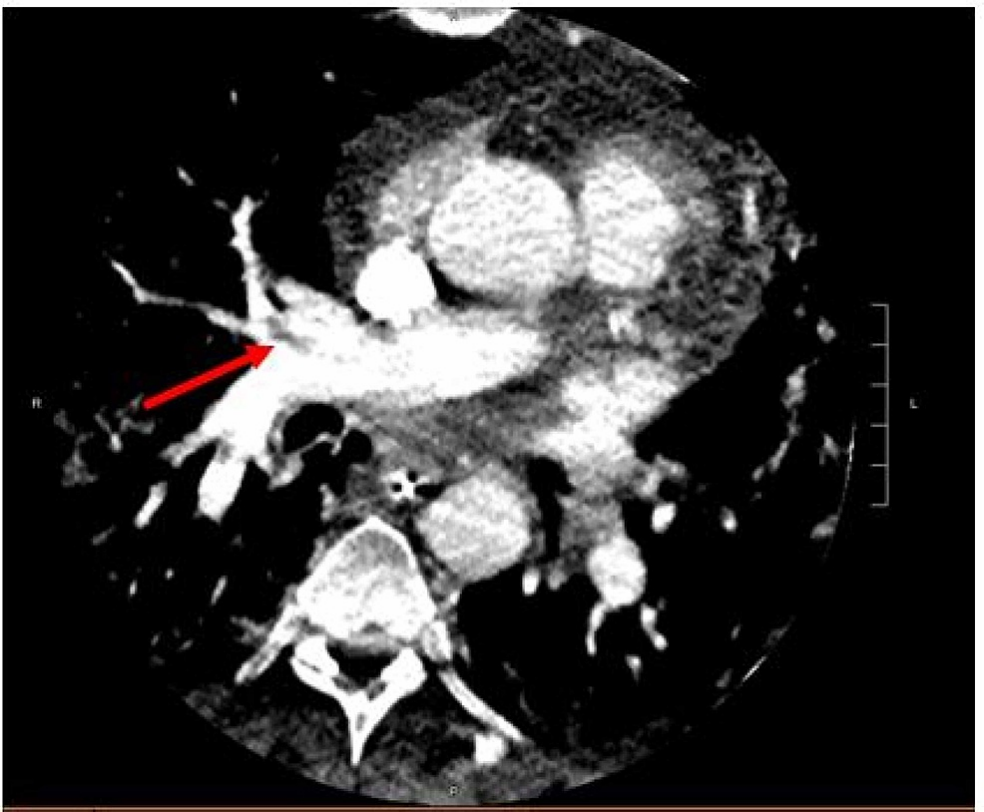
Reconstructed CTA showing upper lobar PE (arrow). CTA, CT angiography; PE, pulmonary embolism

**Figure 3:**
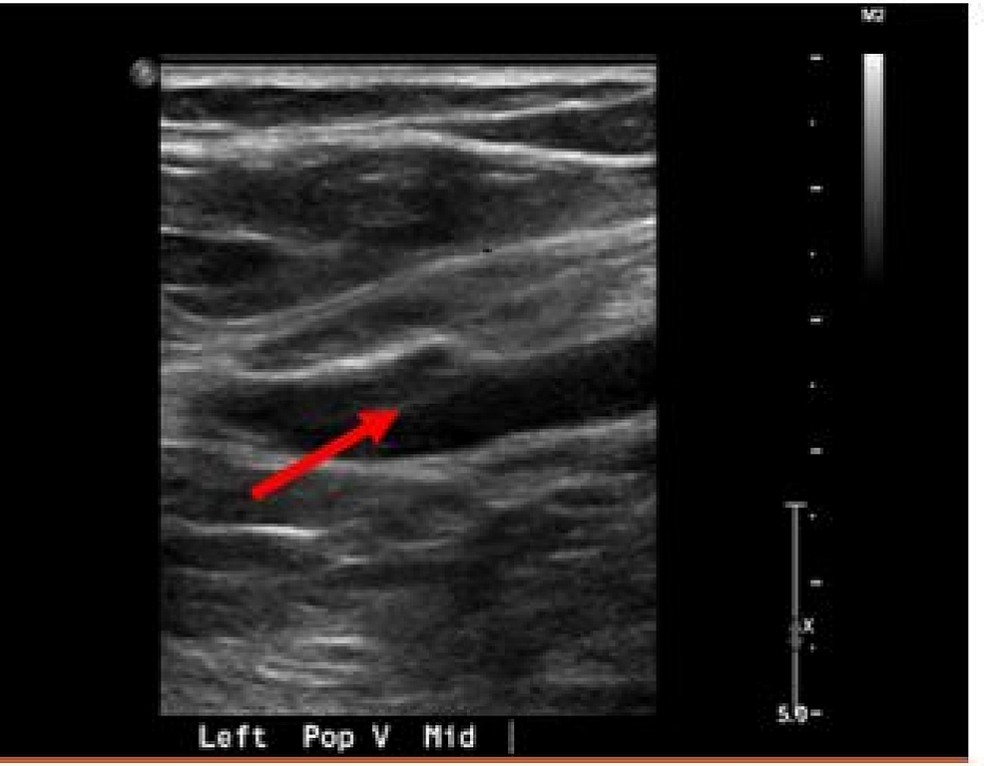
Demonstrating acute DVT in the left popliteal vein. DVT, deep vein thrombosis

Further stroke work-up with cerebral computed tomography angiography (CTA) showed no significant intracranial large vascular occlusion, though the CTA of the neck detected an upper lobe pulmonary embolism (PE) (Figure 2). A lower extremity scan for DVT showed acute left lower extremity DVT (Figure 3). Laboratory workup revealed mild leukocytosis with an elevated white blood cell (WBC) count of 14.4 cells/µL (normal range: 4.1-10.9 cells/µL), a normal hemoglobin level of 13.8 g/dL, and stable platelet counts in the normal range (151,000-250,000 cells/µL). The patient’s inflammatory markers trended down to normal limits, and his persistent leukocytosis was attributed to dexamethasone administration. Liver function, serum electrolytes, and renal function were normal. In the interim, he had an inferior vena cava (IVC) filter placed due to a high risk of a central nervous system (CNS) bleed if he was to be started on therapeutic anticoagulation.

After several days post-stroke, the patient was started on anticoagulation with apixaban and a follow-up CT of the head did not show any neurological changes or significant bleeding. He had subsequent improvement of neurological and respiratory function and was successfully extubated to nasal cannula oxygen and transferred to the general medical floor.

**Figure 4:**
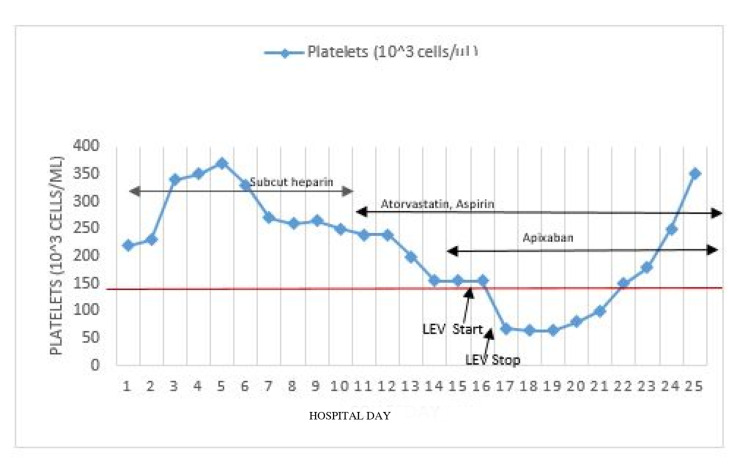
Platelet count as a function of LEV therapy over time. LEV, levetiracetam

**Figure 5:**
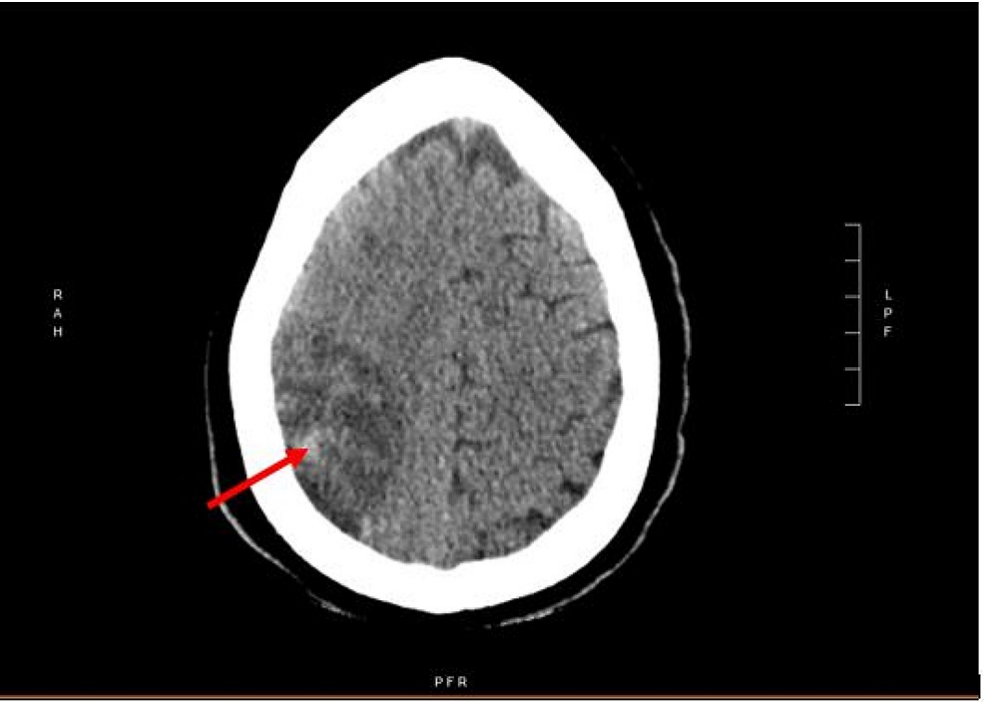
Repeat noncontrast head CT demonstrating an interval sulcal and right basal ganglia infarction and hyper-attenuation (arrow) consistent with petechial hemorrhage.

Three days later, he developed sudden onset tonic-clonic seizures involving the right upper extremity and clenching of teeth which lasted for several minutes and was aborted with a single dose of 4 mg lorazepam followed by a loading dose of IV LEV (2 g). He continued LEV treatment at a maintenance dose of 1 g every 12 hours with good seizure control. His platelet count prior to the initiation of LEV was 151,000 cells/µL but decreased to 68,000 within the next 24 hours and achieved a nadir of 64,000 cells/µL within the following 48 hours (Figure 4). A repeat head CT revealed progressive sulcal and right basal ganglia infarction and hyper-attenuation likely consistent with petechial hemorrhages without overt parenchymal hematoma (Figure 5). 

Considering the patient’s relatively stable hematological profile prior to medication exposure and the temporal relationship between LEV administration and a significant decrease in platelet counts without any other new medication administered, LEV was switched to IV lacosamide at 50 mg twice daily for seizure control. This resulted in a rapid and complete resolution of thrombocytopenia within one week and never required platelet transfusion. With these conservative measures, his hematological profile remained stable but required physical and occupational rehabilitation for his left-sided hemiparesis. He was subsequently discharged to a skilled nursing facility for further rehabilitation.

## Discussion

Drug-induced thrombocytopenia is a well-recognized clinical phenomenon in hospitalized patients which can occur through several mechanisms including direct bone marrow suppression, toxicity, or immune-mediated platelet destruction through the production of anti-platelet antibodies [1, 7-9,13]. LEV, which is a widely used and well-tolerated antiseizure medication, has been rarely associated with this phenomenon, which can be potentially life-threatening in anticoagulated patients due to the potential risk of bleeding as in the index patient [1, 7-12]. Although the exact mechanism of LEV-associated induced thrombocytopenia remains unclear, it has been suggested by previous studies to be immune-mediated, resulting in peripheral platelet destruction [1]. The development of acute significant thrombocytopenia within 24 hours of LEV administration in our patient supports an immune-mediated mechanism of action. Furthermore, the patient's prior history of traumatic brain injury and previous use of several anticonvulsants outside of our health systems, several years prior to the index hospitalization might have predisposed him to LEV exposure and generation of circulating antiplatelet antibodies, which resulted in more robust antibody production and subsequent platelet destruction within 24 hours of drug administration. This rapid onset of platelet destruction occurred earlier than the average duration of 2-20 days reported in a retrospective study conducted by Sahaya et al. [1]. 

Other causes of acute thrombocytopenia considered and ruled out in the index patient include SARS-CoV-2 infection, severe sepsis, liver disease, heparin-induced thrombocytopenia, disseminated intravascular coagulation, and thrombotic micro-angiopathy. Though the patient tested positive for SARS-CoV-2 virus 15 days prior to the thrombocytopenic episode, his resolving pneumonia and respiratory failure, down-trending of inflammatory markers, negative procalcitonin of 0.05 ng/mL (normal range: 0.00-0.10 ng/mL), and normal venous lactate of 1.1 mmol/L (normal range: 0.5-2.0 mmol/L) effectively ruled out persistent SARS-CoV-2 infection and severe sepsis as likely etiologies. His normal liver function ruled out liver disease while his partial thromboplastin time and INR of 1.0 and elevated, but down trending of fibrinogen level from 565 mg/dL (normal range: 159-462 mg/dL) made disseminated intravascular coagulation unlikely. His persistently stable renal function and absence of schistocytes and antibodies to platelet factor IV in peripheral blood effectively ruled out thrombotic microangiopathy and heparin-induced thrombocytopenia, respectively. Also, the observed return of his platelet counts back to baseline following discontinuation of the culprit medication further supports LEV-induced thrombocytopenia. 

Over the past few years, thrombocytopenia or sometimes pancytopenia associated with LEV use have been reported with varying degrees of temporal associations with LEV use [1, 7-12]. In some of these reports, the observed thrombocytopenia was confounded by concurrent use of other medications notable for causing thrombocytopenia or from other conditions [8]. In a retrospective review of 758 hospitalized patients with an age range of 18-93 years, thrombocytopenia occurred in 3.8% (n=29) of patients while on LEV therapy [1]. However, among affected patients, alternative causes of thrombocytopenia were established in 79% (n=23) of patients and about 13% (n=4) had pre-existing thrombocytopenia without significant change in platelet count after the commencement of LEV. Thrombocytopenia was noted to be dose-related in one patient, and the pathophysiology was also suggested to be immune-mediated based on the timing of improvements in platelet counts upon discontinuation of LEV [1]. 

The reversibility of thrombocytopenia with the withdrawal of LEV as occurred in our patient was also noted in other case reports and retrospective review of this phenomenon [1, 7-12]. Further analysis of adverse drug event reports for LEV is necessary to determine whether there is a synergistic effect or an increased risk of LEV-induced thrombocytopenia in patients on a regimen of anticoagulants.

## Conclusions

Levetiracetam can induce acute thrombocytopenia which can be clinically significant in the setting of anticoagulation due to increased risk of bleeding. Close monitoring of platelet count and continued assessment of bleeding risk are warranted for such patients.
